# Outpatient Tinnitus Clinic, Self-Help Web Platform, or Mobile Application to Recruit Tinnitus Study Samples?

**DOI:** 10.3389/fnagi.2017.00113

**Published:** 2017-04-21

**Authors:** Thomas Probst, Rüdiger C. Pryss, Berthold Langguth, Myra Spiliopoulou, Michael Landgrebe, Markku Vesala, Stephen Harrison, Johannes Schobel, Manfred Reichert, Michael Stach, Winfried Schlee

**Affiliations:** ^1^Georg-Elias-Müller Institute for Psychology, Georg-August University GöttingenGöttingen, Germany; ^2^Institute of Databases and Information Systems, Ulm UniversityUlm, Germany; ^3^Department of Psychiatry and Psychotherapy of the University of Regensburg at Bezirksklinikum Regensburg, University of RegensburgRegensburg, Germany; ^4^Department of Technical and Business Information Systems, Otto-von-Guericke-University MagdeburgMagdeburg, Germany; ^5^Clinic Lech-MangfallAgatharied, Germany; ^6^Tinnitus Hub LtdHemsworth, UK

**Keywords:** tinnitus, recruitment, crowdsourcing, crowdsensing, clinical data

## Abstract

For understanding the heterogeneity of tinnitus, large samples are required. However, investigations on how samples recruited by different methods differ from each other are lacking. In the present study, three large samples each recruited by different means were compared: *N* = 5017 individuals registered at a self-help web platform for tinnitus (crowdsourcing platform Tinnitus Talk), *N* = 867 users of a smart mobile application for tinnitus (crowdsensing platform TrackYourTinnitus), and *N* = 3786 patients contacting an outpatient tinnitus clinic (Tinnitus Center of the University Hospital Regensburg). The three samples were compared regarding age, gender, and duration of tinnitus (month or years perceiving tinnitus; subjective report) using chi-squared tests. The three samples significantly differed from each other in age, gender and tinnitus duration (*p* < 0.05). Users of the TrackYourTinnitus crowdsensing platform were younger, users of the Tinnitus Talk crowdsourcing platform had more often female gender, and users of both newer technologies (crowdsourcing and crowdsensing) had more frequently acute/subacute tinnitus (<3 months and 4–6 months) as well as a very long tinnitus duration (>20 years). The implications of these findings for clinical research are that newer technologies such as crowdsourcing and crowdsensing platforms offer the possibility to reach individuals hard to get in contact with at an outpatient tinnitus clinic. Depending on the aims and the inclusion/exclusion criteria of a given study, different recruiting strategies (clinic and/or newer technologies) offer different advantages and disadvantages. In general, the representativeness of study results might be increased when tinnitus study samples are recruited in the clinic as well as via crowdsourcing and crowdsensing.

## Introduction

Tinnitus is characterized by the perception of a sound without a corresponding external sound source (Baguley et al., 2013; Langguth et al., 2013). A recent review on prevalence rates of tinnitus in 16 countries found that between 5.1% and 42.7% of the population report tinnitus (McCormack et al., 2016). The prevalence rates typically vary depending on the age, the birth cohort (Nondahl et al., 2012; Martinez et al., 2015), and the definition of tinnitus used in the epidemiological study (McCormack et al., 2016). A recent US study found that 9.6% of Americans experienced tinnitus in the last year and that only 49.4% discussed the tinnitus with a physician (Bhatt et al., 2016). In case tinnitus patients seen by physicians are not representative for the whole sample of individuals perceiving tinnitus, the representativeness of studies recruiting patients only in medical practices or hospitals might be limited. In the area of anxiety and depression, for example, researchers found differences between patients contacting an outpatient clinic, patients being treated in an Internet-based clinic, and individuals with anxiety or depressive disorders from a national epidemiological survey (Titov et al., 2010). Another study on Internet-based psychotherapy for depression found that patients recruited by online advertisements differed from patients recruited by newspaper advertisements in demographics and depression severity (Lindner et al., 2015).

In the case of tinnitus, several patient databases have been established with the aim to better understand the heterogeneity of tinnitus (Meikle, 1997; Landgrebe et al., 2010; Witsell et al., 2011). However, all these databases collect data from patients who present themselves at a tinnitus clinic. For investigating tinnitus heterogeneity, it is of high importance to know how samples recruited by different methods vary and which recruitment methods are most efficient for the recruitment of specific tinnitus subgroups.

Although the recruitment of individuals not contacting physicians is traditionally difficult, online-recruitment has been shown to be promising in clinical and health science to overcome this obstacle (e.g., Morgan et al., 2013; Bevelander et al., 2014; Gioia et al., 2016; Kayrouz et al., 2016; Thornton et al., 2016; Topolovec-Vranic and Natarajan, 2016). In a recent review on online-recruitment in health studies, Lane et al. (2015) stated, though, that “more empirical evidence is needed to make specific recommendations” (p. 1). The claim for more empirical research might be especially relevant for the relatively new participant-led research paradigm (Vayena and Tasioulas, 2013) including crowdsourcing (e.g., Swan, 2012; Ranard et al., 2014; Chandler and Shapiro, 2016) and crowdsensing (e.g., Ganti et al., 2011; Guo et al., 2014, 2015). “Crowdsourcing is a type of participative online activity in which an individual, an institution, a non-profit organization, or company proposes to a group of individuals of varying knowledge, heterogeneity, and number, via a flexible open call, the voluntary undertaking of a task. The undertaking of the task, of variable complexity and modularity, and in which the crowd should participate bringing their work, money, knowledge and/or experience, always entails mutual benefit. The user will receive the satisfaction of a given type of need, be it economic, social recognition, self-esteem, or the development of individual skills, while the crowdsourcer will obtain and utilize to their advantage what the user has brought to the venture, whose form will depend on the type of activity undertaken” (Estellés-Arolas and González-Ladrón-de-Guevara, 2012, p. 197). Guo et al. (2015) defined mobile crowdsensing and computing (MCSC) as follows: “MCSC extends the vision of participatory sensing by leveraging both participatory sensory data from mobile devices (offline) and user-contributed data from mobile social networking services (online). Further, it explores the complementary roles and presents the fusion/collaboration of machine and human intelligence in the crowdsensing and computing processes” (Guo et al., 2015, p. 1). In contrast to crowdsourcing, crowdsensing relies solely on mobile technology and integrates sensors to collect data, for example, behavioral (e.g., physical activity level, gait pattern), physiological (e.g., heart rate, electrodermal activity), and environmental (e.g., environmental sound level, GPS location) variables.

As it remains unclear whether online-recruitment by crowdsourcing and crowdsensing offers the potential to reach individuals with tinnitus that are different from the ones directly consulting a physician, the study at hand compared tinnitus patients visiting an outpatient tinnitus clinic, users of a tinnitus crowdsourcing platform (moderated self-help web platform) and users of a tinnitus crowdsensing platform (mobile application).

## Materials and Methods

### Samples

The following three samples were investigated:

1. Tinnitus crowdsourcing sample “Tinnitus Talk”: Tinnitus Hub^1^ operates the moderated self-help platform Tinnitus Talk^2^. The platform was established in March 2011 by Markku Vesala. With 16,500 registered participants (as of December 2016) and over 210,000 unique readers every month, Tinnitus Talk is one of the most active tinnitus-dedicated platforms for information delivery, experience exchange, and self-help among individuals with tinnitus. The data of the present study relies on a survey ran from February 8th till March 13th 2016.
2. Tinnitus crowdsensing sample “TrackYourTinnitus”: TrackYourTinnitus (TYT; Pryss et al., 2015a,b)^3^ is an application for mobile-devices (iOS and Android) that allows the tracking of tinnitus in daily life by ecological momentary assessments. Although monitoring tinnitus repeatedly directs the attention towards the tinnitus, Schlee et al. (2016) showed that using TYT does not deteriorate the tinnitus. Other studies on TYT investigated the role of emotional states (Probst et al., 2016a) as well as emotion dynamics (Probst et al., 2016b) in tinnitus. The data presented here was collected from April 2014 to June 2016.
3. Outpatient tinnitus clinic sample “Tinnitus Center Regensburg”: The University Hospital Regensburg hosts a tinnitus center^4^, established 2007, and having about 300 tinnitus patients per annum. To date (December 2016), the Tinnitus database encompasses medical records for about 3000 patients.

At the Tinnitus Center Regensburg, patients gave written informed consent that data were gathered and analyzed for the Tinnitus Research Initiative Database, which was approved by the Ethics Committee of the University Hospital of Regensburg. The material and the methods of TrackYourTinnitus were approved by the Ethics Committee of the University Hospital Regensburg and were carried out in accordance with the approved guidelines; written consent, however, was not possible to obtain for the users of TrackYourTinnitus. Information that the data will be used for scientific analyses is included in the mobile applications as well as on the website and, therefore, the TrackYourTinnitus users were informed that the data will be used for scientific purposes. Written informed consent was also not possible to obtain for the users of Tinnitus Hub/Tinnitus Talk, but the “Terms and Rules” of the website informed the users that the collected data will be analyzed for scientific purposes. All the data were saved anonymously.

### Measures

Three variables were assessed in the three samples by self-reports: age, gender, and duration of tinnitus (month or years perceiving tinnitus; subjective report). In the Tinnitus Talk survey, the variables age (<18 years, 18–24 years, …; see Table 1) and tinnitus duration (<3 months, 4–6 months, …; see Table 1) were assessed in categories. Therefore, the metric scores of age and tinnitus duration as assessed in the Tinnitus Center Regensburg and TrackYourTinnitus were categorized accordingly to be able to perform statistical analyses.

**Table 1:** **Description of the three samples and comparisons between the samples**.

	Tinnitus Talk	TrackYourTinnitus	Tinnitus Center Regensburg	Between-group statistics
Total number	5017	867	3786	
**Age in years**				*X*^2^ = 366.7;
% <18	1.0	1.6	0.5	*p* < 0.001
% 18–24	5.4	4.4	2.4	
% 25–34	11.4	20.7	6.6	
% 35–44	13.6	22.5	15.7	
% 45–55	20.9	26.5	29.6	
% 55–64	30.2	18.9	26.7	
% 65–74	15.1	5.2	15.5	
% >75	2.4	0.3	3.1	
**Gender**				*X*^2^ = 103.5;
% female	42.8	27.1	35.1	*p* < 0.001
% male	57.2	72.9	64.9	
**Tinnitus duration**				*X*^2^ = 393.7;
% <3 months	6.4	14.2	2.1	*p* < 0.001
% 4–6 months	5.7	6.8	3.4	
% 6–12 months	10.1	7.0	9.3	
% 1–2 years	14.7	7.6	16.2	
% 3–5 years	13.1	15.4	20.6	
% 5–10 years	18.3	16.0	17.1	
% 10–20 years	15.3	16.9	20.7	
% >20 years	16.4	16.1	10.6	

### Statistical Analysis

Chi-squared tests were performed with R and the significance value was set to *p* < 0.05.

## Results

The three samples comprised *N* = 9670 individuals. Information on age was available for *n* = 8766 individuals, information on gender for *n* = 9607, and information on tinnitus duration for *n* = 8409. Full descriptions of the samples are presented in Table 1.

### Age

**Figure 1:**
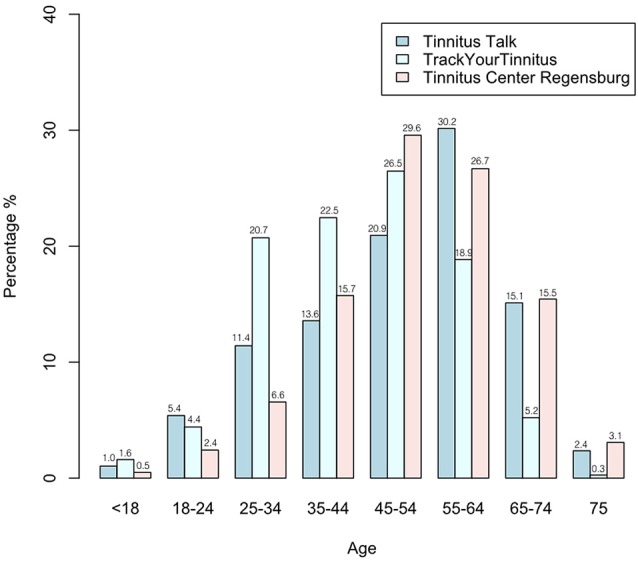
**Distribution of age in the three samples**.

A chi-squared test was calculated to test whether the distribution of age (see Table 1; Figure 1) is different between the samples. With an *X*^2^ = 366.7, the hypothesis that the samples had an equal age distribution was rejected (*p* < 0.001). The most obvious differences emerged in the percentage of individuals with an age between 25 and 44 years as well as in the percentage of individuals with an age between 55 and 74 years: The 25–44 years aged individuals were more often among the TrackYourTinnitus users (25–34 years: 20.7%; 35–44 years: 22.5%) than among the Tinnitus Talk users (25–34 years: 11.4%; 35–44 years: 13.6%) and among the patients of the Tinnitus Center Regensburg (25–34 years: 6.6%; 35–44 years: 15.7%). Yet, the 55–74 years aged adults were more frequently among the Tinnitus Talk users (55–64 years: 30.2%; 65–74 years: 15.1%) and among the patients at the Tinnitus Center Regensburg (55–64 years: 26.7%; 65–74 years: 15.5%) than among the TrackYourTinnitus users (55–64 years: 18.9%; 65–74 years: 5.2%).

### Gender

**Figure 2:**
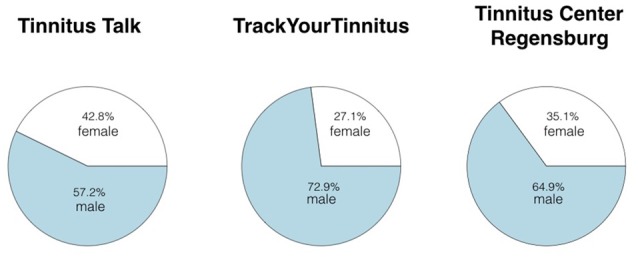
**Distribution of gender in the three samples**.

A chi-squared test was also calculated to investigate whether the gender distribution of the three sample groups is equal or different (see Table 1; Figure 2). Based on an *X*^2^ = 103.5, the hypothesis of an equal gender distribution was rejected (*p* < 0.001). Although the participants were more frequently men than women in all three samples, TrackYourTinnitus had the lowest rate of females (27.1%) and Tinnitus Talk the highest (42.8%).

### Tinnitus Duration

**Figure 3:**
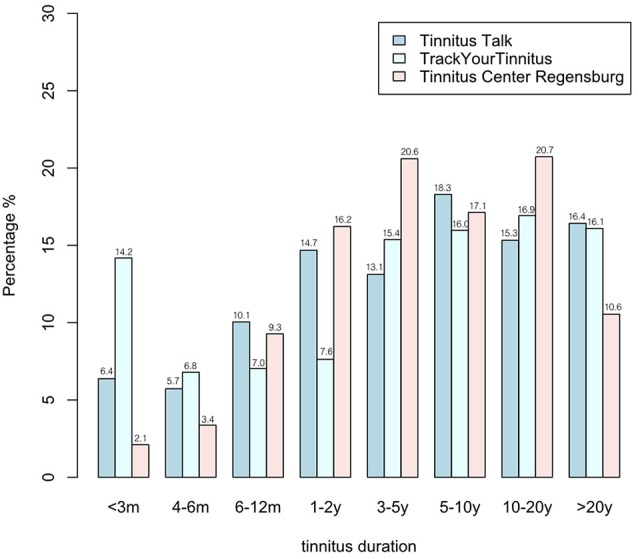
**Distribution of tinnitus duration in the three samples**.

Again a chi-squared test was calculated to test whether the distribution of tinnitus duration is equal or different between the three samples (see Table 1; Figure 3). With an *X*^2^ of 393.7, the hypothesis of an equal tinnitus duration distribution was rejected (*p* < 0.001). Differences were apparent for individuals with a tinnitus duration <3 months: 14.2% of the TrackYourTinnitus users were in this category, but only 6.4% of the Tinnitus Talk users and only 2.1% of the Tinnitus Center Regensburg patients. However, TrackYourTinnitus users reported less often 1–2 years of tinnitus duration (7.6%) than Tinnitus Talk users (14.7%) and patients at the Tinnitus Center Regensburg (16.2%). The Tinnitus Center Regensburg patients reported more often 3–5 years and 10–20 years of tinnitus duration (3–5 years: 20.6%; 10–20 years: 20.7%) than Tinnitus Talk users (3–5 years: 13.1%; 10–20 years: 15.3%) and TrackYourTinnitus users (3–5 years: 15.4%; 10–20 years: 16.9%). The individuals with tinnitus duration >20 years, however, were more frequently among the Tinnitus Talk users (16.4%) and the TrackYourTinnitus users (16.1%) than among the patients at the Tinnitus Center Regensburg (10.6%).

## Discussion

The study at hand compared individuals contacting an outpatient tinnitus clinic (Tinnitus Center Regensburg), individuals registered at a self-help web platform (crowdsourcing platform Tinnitus Talk) and users of an application for mobile devices (crowdsensing platform TrackYourTinnitus). The aim of the study was to investigate whether newer technologies (crowdsourcing and/or crowdsensing) offer possibilities for future studies to reach individuals with tinnitus that are different from the ones directly contacting an outpatient clinic. In summary, we found that the samples differed in the investigated variables. The result that patients of an outpatient clinic differed from users of newer technologies is in line with a study on anxiety and depression comparing patients of an outpatient clinic with those attending an Internet clinic (Titov et al., 2010). Moreover the results of the current study correspond with a recent review (Topolovec-Vranic and Natarajan, 2016), which reported that populations recruited by traditional methods were not comparable to samples recruited by newer technologies (the review focused on social media) in most of the studies (12 vs. 2).

One of the variables investigated in the present study was age. The results revealed that younger individuals (≤44 years) used more frequently the crowdsensing platform for tinnitus, whereas older individuals (≥55 years) were more often among the users of the crowdsourcing platform for tinnitus as well as among the patients of an outpatient tinnitus clinic. Thus, crowdsensing might be potent to recruit younger individuals but not as suited for the recruitment of older individuals. Nevertheless, crowdsensing might be appropriate to recruit older individuals in the future as the younger generation (which now uses crowdsensing) becomes older.

Another evaluated variable in the current study was gender. Gender was predominately male in all three samples and this result is in line with previous research on tinnitus: “In fact, most previous studies, but not all … showed higher tinnitus prevalence in men than in women” (Gallus et al., 2015; p. 16). The crowdsourcing platform Tinnitus Talk had the highest percentage of female individuals, whereas the crowdsensing platform TrackYourTinnitus had the lowest. Therefore, crowdsourcing might be more appropriate than crowdsensing when aiming to recruit women by newer technologies.

The third investigated variable in the study at hand was tinnitus duration (month or years perceiving tinnitus; subjective report). Duration of tinnitus is clinically and scientifically relevant to define acute, subacute and chronic tinnitus. The results of the current study revealed that acute and subacute tinnitus are more frequent among the users of crowdsourcing and crowdsensing platforms than among the patients of an outpatient clinic. Long waiting times for appointments in specialized tinnitus clinic might contribute to this effect. Regardless the reasons for the delay in seeing a specialist, our data indicate that individuals with tinnitus search for information or help in the Internet already early after symptom onset. As interventions are already helpful in the acute and subacute stages of tinnitus (Nyenhuis et al., 2013b) and might prevent a chronic course, offering helpful interventions for individuals with acute and subacute tinnitus is crucial. As more of these individuals can be reached by crowdsourcing or crowdsensing platforms than by an outpatient clinic, the implementation of appropriate self-help interventions in crowdsourcing or crowdsensing platforms might be promising (e.g., Heron and Smyth, 2010; Donker et al., 2013). The finding that Internet-based self-help is as effective as face-to-face interventions for tinnitus patients (e.g., Nyenhuis et al., 2013a; Jasper et al., 2014; Andersson, 2015) supports this line of argumentation. Another interesting result in the context of tinnitus duration was that individuals who perceived their tinnitus for a very long time (>20 years), were more frequent among the crowdsourcing and crowdsensing samples than among the sample of the outpatient clinic. It could be speculated that these individuals experienced past treatments as ineffective and consequently gave up seeking medical or psychological help (learned helplessness; Abramson et al., 1978) or have already established effective coping strategies. These individuals will not contact a physician or a clinic, although interventions, which these individuals have not tried yet, might be helpful. These findings at least suggest that it might be useful to provide information about interventions and current innovative treatment approaches in crowdsourcing or crowdsensing platforms.

One limitation of the study at hand is that we analyzed only one crowdsourcing platform for tinnitus, only one crowdsensing platform for tinnitus, and only one outpatient tinnitus clinic. Although our samples are relatively large, even more representative results might be obtained by multicenter studies including several clinics and several crowdsourcing (two crowdsourcing platforms were investigated, for example, by Briones and Benham, 2017) as well as several crowdsensing platforms. Another shortcoming of the crowdsourced and crowdsensed data is that the robustness and accuracy of the online-collected data is difficult to verify. Previous studies could, however, provide support for the trustworthiness of web-based research (e.g., Meyerson and Tryon, 2003; Gosling et al., 2004). Moreover comparisons with data from population-based epidemiological studies (see Titov et al., 2010) would be desirable to identify how representative the three samples of the current study are for the total tinnitus population. We abstained from such a comparison, since data from available epidemiological studies are highly variable (McCormack et al., 2016). Finally, since clinical variables were not assessed in all three samples, we could not compare the three samples regarding clinical characteristics of tinnitus (e.g., tinnitus severity). Tinnitus severity is usually important in the recruitment process of clinical studies and usually is measured by psychometrically sound instruments such as the “Tinnitus Handicap Inventory” (THI; Newman et al., 1996). Those who use an app or online site may have less bothersome tinnitus and may have no intention of seeking medical attention. At least some may be just curious but not need any treatment or intervention. Comparing the severity of these groups is an important task for future research to clarify whether recruiting individuals with tinnitus using a tinnitus app or website will aid our understanding of bothersome tinnitus. Such further studies will aid to find the best recruitment strategy for a given study.

Despite these limitations, the current study is the first one comparing tinnitus patients of an outpatient clinic with users of crowdsourcing and crowdsensing platforms for tinnitus. We showed that these newer technologies offer promising perspectives to reach individuals hard to get in contact with at an outpatient tinnitus clinic (e.g., individuals with acute/subacute tinnitus, younger individuals, as well as individuals perceiving tinnitus for a very long time).

## Author Contributions

TP substantially contributed to the design of the study and data preparation, drafted and revised the manuscript. RCP substantially contributed to the design of the study, data preparation, the TrackYourTinnitus platform and revised the manuscript. BL substantially contributed to the design of the study, data collection at the Tinnitus Center Regensburg, the TrackYourTinnitus platform and revised the manuscript. MS substantially contributed to the design of the study and revised the manuscript. ML substantially contributed to the data collection at the Tinnitus Center Regensburg and revised the manuscript. MV and SH substantially contributed to the Tinnitus Talk platform, and revised the manuscript. JS, MR and MS substantially contributed to the TrackYourTinnitus platform, and revised the manuscript. WS substantially contributed to the design of the study, the TrackYourTinnitus platform, drafted and revised the manuscript, and performed the statistical analyses.

## Conflict of Interest Statement

The authors declare that the research was conducted in the absence of any commercial or financial relationships that could be construed as a potential conflict of interest. The reviewer TLE and handling Editor declared their shared affiliation, and the handling Editor states that the process nevertheless met the standards of a fair and objective review.

## Footnotes

^1^www.tinnitushub.com

^2^www.tinnitustalk.com

^3^www.trackyourtinnitus.org

^4^www.tinnituszentrum-regensburg.de
